# *Lactobacillus reuteri* and *Staphylococcus aureus* differentially influence the generation of monocyte‐derived dendritic cells and subsequent autologous T cell responses

**DOI:** 10.1002/iid3.115

**Published:** 2016-07-29

**Authors:** Yeneneh Haileselassie, Marit Navis, Nam Vu, Khaleda Rahman Qazi, Bence Rethi, Eva Sverremark‐Ekström

**Affiliations:** ^1^ Department of Molecular Biosciences, The Wenner‐Gren InstituteStockholm UniversityStockholmSweden; ^2^ Department of MedicineKarolinska University HospitalStockholmSweden

**Keywords:** Cytokine, dendritic cells, *Lactobacillus reuteri*, monocytes, PCR array, *Staphylococcus aureus*, T cell

## Abstract

**Introduction:**

In early‐life, the immature mucosal barrier allows contact between the gut microbiota and the developing immune system. Due to their strategic location and their ability to sample luminal antigen, dendritic cells (DC) play a central role in the interaction of microbes and immune cells in the gut. Here, we investigated how two bacteria associated with opposite immune profiles in children, that is, *Lactobacillus* (*L*.) *reuteri* and *Staphylococcus* (*S*.) *aureus*, influenced the differentiation of monocytes in vitro as well how the generated DC impacted T cell responses.

**Methods:**

We exposed monocyte cultures to cell‐free supernatants (CFS) from these bacteria during their differentiation to DC.

**Results:**

The presence of *L. reuteri*‐CFS during DC differentiation resulted in DC with a more mature phenotype, in terms of up‐regulated surface markers (HLA‐DR, CD86, CD83, CCR7) and enhanced cytokine production (IL6, IL10, and IL23), but had a reduced phagocytic capacity compared with non‐treated monocyte‐derived DC (Mo‐DC). However, upon LPS activation, *L. reuteri*‐CFS‐generated DC displayed a more regulated phenotype than control Mo‐DC with notable reduction of cytokine responses both at mRNA and protein levels. In contrast, *S. aureus*‐CFS‐generated DC were more similar to control Mo‐DC both without and after LPS stimulation, but they were still able to induce responses in autologous T cells, in the absence of further T cell stimulation.

**Conclusions:**

We show that bacterial signals during DC differentiation have a profound impact on DC function and possibly also for shaping the T cell pool.

## Introduction

The gut microbiota plays a critical role in modulating host physiology, including immunity 1. This is most notable at early age, as the development of the intestinal mucosa continues during the postnatal period, and the immature barrier allows contact between the gut microbiota and the developing immune system 2, 3. An altered colonization of the gut may lead to improper development of the infant immune system, with an increased risk of developing immune‐mediated diseases, for example, allergy, later in life 4, 5, 6.

How gut microbes influence immunity and the development of immune‐mediated diseases is still an enigma, but it is highly likely that factors released from gut microbes interact with key intestinal immune cells 7, 8. Due to their strategic location in the gut and their ability to sample luminal antigen, dendritic cells (DC) play a central role in the interaction of microbes and immune cells 9. The majority of studies involving gut DC development are based on mouse models; research concerning human gut DC development is very limited, primarily due to practical constraints in studying human tissue and harvesting human gut DC. Therefore, the most common method to study human DC is by generating them from circulatory monocytes treated with GM‐CSF and IL4. Although some studies suggest that this is an appropriate model to study DC 10, 11, 12, it is now evident that DC mainly arise from DC‐committed precursors (pre‐DC) at steady state 13, 14, 15, but there are also studies suggesting that they can arise also from monocytes. Still, under inflammatory conditions, it is clear that DC will develop from monocytes also in the gut 16, 17.

In previous work from our group, we have made several observations that together point toward a significant impact of lactobacilli and *Staphylococcus (S.) aureus* on allergy development and/or immune function during early life in humans 18, 19, 20, 21. While an early‐life gut microbiota positive for lactobacilli was associated with a regulated immunological profile and protection against allergy development in children, early colonization with *S. aureus* was associated with strong in vitro immune responses, particularly when lactobacilli were not detected. These associations between in vivo colonization and immune responsiveness in vitro have been confirmed in further studies—and we have observed that while different lactobacilli (e.g., *Lactobacillus* [*L*.] *reuteri and L. rhamnosus*) readily activate innate immune cells like monocytes, they are capable of dampening *S. aureus*‐induced immune activation of adaptive immune cells like T cells in vitro 22. Recently, we have also shown that *L. reuteri* has a significant impact on (the maturation of) both conventional and retinoic acid‐derived DC in making them less responsive to an inflammatory challenge 23. To further explore how gut bacteria can influence immunity in young children, we sought to understand how two bacteria associated with dramatically opposite immune profiles in children, *L. reuteri* and *S. aureus*, influence the differentiation of monocytes in vitro as well as subsequent effects on the bacteria‐generated DC in regulating T cell cytokine production in the absence of T cell‐specific antigens.

## Materials and Methods

### Ethical permission

All experiments were approved by the Regional Ethics Committee in Stockholm (Dnr 2014/2052‐32). All study subjects gave their informed written consent and all samples were coded and stored and used as stated in the approved ethical application. It is not possible to connect published data to any individual.

### Generation of cell‐free supernatants (CFS) from *L. reuteri* DSM 17938 and *S. aureus* 161:2

*Lactobacillus reuteri* DSM 17938 (Biogaia AB, Stockholm, Sweden) and *S. aureus* 161:2 were kindly provided by Stefan Roos, the Swedish University of Agricultural Sciences and Åsa Rosengren, (The National Food Agency, Uppsala, Sweden), respectively. *Lactobacillus reuteri* DSM 17938 was cultured in MRS broth (Oxoid, Hampshire, UK) at 37°C for 20 h, while *S. aureus* 161:2 was cultured in BHI broth (Merck, Darmstadt, Germany) at 37°C for 72 h (both in still culture). The CFS were removed from the bacterial pellets by centrifugation at 14,000*g*. The CFS were sterile‐filtered (0.2 μm) and stored at −20°C until used. Before stimulation of DC, the *L. reuteri*‐CFS was diluted 1:1 with HEPES to neutralize the pH 22.

### Differentiation of CD14^+^ monocytes to DC in the presence of bacteria‐CFS

Peripheral blood mononuclear cells (PBMC) were isolated from buffy coats from healthy blood donors using Ficoll (GE Healthcare, Logan, UT) gradient centrifugation. All experiments were approved by the Regional Ethics Committee in Stockholm (Dnr 2014/2052‐32). All study subjects gave their informed written consent and all samples were coded and stored and used as stated in the approved ethical application. It is not possible to connect published data to any individual.

CD14^+^ monocytes were enriched by negative selection using EasySep™ human monocyte enrichment kits (STEMCELL™ Technologies, Grenoble, France). A 4 × 10^5^ cells/mL of the enriched monocytes were seeded in 6‐well plates in complete culture medium (RPMI‐1640 [GE Healthcare], 10% heat‐inactivated fetal bovine serum [Gibco‐Invitrogen, Waltham, MA], 1% l‐glutamine [GE Healthcare], 1% penicillin‐streptomycin [Thermo Scientific, Waltham, MA], 2% sodium pyruvate [Gibco‐Invitrogen], 2% HEPES buffer [GE Healthcare], and 50 µM β‐mercaptoethanol [Sigma–Aldrich, St. Louis, MO]), supplemented by 25 ng/mL rHuGM‐CSF and 20 ng/mL rHuIL4 (both from Peprotech, Inc., Rocky Hill, NJ).

On day 3, the cultures were treated with 5% *L. reuteri*‐CFS (LR‐DC) and/or 5% *S. aureus* 161:2‐CFS (LR + SA‐DC and SA‐DC, respectively) diluted in complete culture media, and kept in culture for additional three days. As a control, cells were kept in culture medium alone (Mo‐DC). On day 6, culture supernatants were collected and stored at −20°C until further analysis. The cells were used for phenotyping or further stimulation.

### Phagocytosis assay

To assess the phagocytic ability of the DC generated in the presence of bacteria, the CytoSelectTM 96‐Well Phagocytosis Assay was performed (Cell Biolabs, San Diego, CA). Briefly, the DC were incubated with enzyme‐labeled *Escherichia* (*E*.) *coli* particles for 5 h at 37°C 5% CO_2_. After washing and blocking, cells were permeabilized and incubated with substrate for 20 min. Then, the reaction was stopped and the optical density (OD) value was measured with a spectrophotometer (Molecular Devices Corp, Sunnyvale, CA). Each sample, including a negative control without *E. coli* particles to correct for background color, was assayed in duplicate. Cells pre‐treated for 30 min with 10 µM cytochalasin D, a phagocytosis inhibitor, were included as a negative control.

### Activation of the bacteria‐CFS‐conditioned DC with LPS

On day 6, LR‐DC, SA‐DC, LR + SA‐DC, and Mo‐DC were collected, washed, and reseeded at 1 × 10^6^ cells/mL and further stimulated with ultrapure lipopolysaccharide (LPS)‐0111:B4 (500 ng/mL) (InvivoGen, San Diego, CA) for 24 h. As a control, cells were kept in culture medium. Culture supernatants from the 24‐h stimulations were collected and stored at −20°C until further analysis. The cells were used for surface staining.

### RT^2^ Profiler™ PCR array of the LPS‐stimulated DC

Gene expression profile of LR‐DC, SA‐DC, LR + SA‐DC, and Mo‐DC were investigated by using the RT^2^ Profiler™ PCR Array Human Dendritic and Antigen Presenting Cell (PAHS‐406ZF) (Qiagen, Germantown, MD) following stimulation with LPS for 24 h. A 1.50 × 10^6^ Stimulated DC cells were homogenized with QIAshredder^®^ (Qiagen) and total RNA was isolated with the RNeasy^®^ Mini Kit (Qiagen). The amount, purity, and quality of the generated RNA were evaluated using a NanoDrop™ 8‐sample spectrophotometer (Thermo Scientific) and an Agilent 2100 Bioanalyzer (Agilent Technologies, Santa Clara, CA). Only RNA samples with a RNA integrity number (RIN) >8.5 were used for gene expression analysis. The RT^2^ First Strand Kit (Qiagen) was used to generate cDNA, starting with 0.5 μg of total RNA for each sample. Quantitative polymerase chain reactions were performed using the RT^2^ SYBR Green ROX qPCR Mastermix as recommended by the manufacturer (Qiagen), and the array was run with the LightCycler^®^ 480 real‐time PCR (Roche Applied Science, Penzberg, Germany). Results were analyzed in the web‐based GeneGlobe Data Analysis Center (Qiagen). Gene expression levels were normalized to the reference genes: actin‐beta (ACTB) and glyceraldehydes‐3‐phosphate dehydrogenase (GAPDH). The reference genes were selected based on their stability in expression among samples (C_T_ difference <1).

### Staining and flow cytometry analysis of bacteria‐conditioned DC pre‐ or post‐stimulation with LPS

LR‐DC, SA‐DC, LR + SA‐DC, and Mo‐DC, prior to or post‐stimulation with LPS, were stained with the following monoclonal antibodies: CD14 FITC‐A (clone M5E2), CD86 PerCP‐A (clone IT2.2), CD83 PE‐Cy7‐A (clone HBI5e), CD11c APC‐A (clone B‐ly6), HLA‐DR PerCP‐A (clone G46‐6), DC‐SIGN BV421‐A (clone DCN46) (all from BD Biosciences, San Jose, CA), and CCR7 BV421‐A (clone G043H7) (BioLegend, San Diego, CA). The FACSVerse instrument and the FACS Suite software (BD Biosciences) were used to acquire data. Corresponding isotype‐matched antibodies were used as negative controls. Detailed gating strategies for the DC can be found in Supplementary Figure S1A. The results show either the percentage of positive cells within a given population or the mean surface expression of receptors per cell defined as geometrical mean fluorescence intensity (MFI). Analysis was done with FlowJo Software (TreeStar, Ashland, OR) and Cytobank (Cytobank, Inc., Mountain View, CA).

### T cell co‐culture with LR‐DC, SA‐DC, LR + SA‐DC, and Mo‐DC pre‐stimulated with LPS or bacteria‐CFS

On day 6, LR‐DC, SA‐DC, LR + SA‐DC, and Mo‐DC were stimulated for 5 h with LPS. In some experiments, the Mo‐DC were also further stimulated with 5% *L. reuteri*‐CFS and or 5% *S. aureus* 161:2‐CFS for 5 h. As a control, cells were kept in culture medium. Prior to co‐culture with T cells, the stimulated DC were washed three times with complete culture medium, to prevent carry‐over of bacterial‐factor to the co‐culture. Autologous T cells were negatively selected from PBMC with the EasySep™ Human T Cell Enrichment Kit (STEMCELL™ Technologies) according to the manufacturer's instructions. T cell purity was 95.9% as determined by flow cytometry. DC and T cells were co‐cultured in a 1:4 ratio (DC:T cell) for 24 h without additional stimuli. The cells were used for flow cytometry analysis. Culture supernatants from the 24‐h stimulations of monensin untreated cell culture were collected and stored at −20°C until further analysis with ELISA.

### Proliferation assay

For measuring proliferation, T cells were labeled at 37°C, 5% CO_2_ with 5 µM CellTrace™ Violet (Molecular probe, Life Technologies, Eugene, OR) for 20 min. Labeled T cells were co‐cultured with bacteria CFS‐pre‐treated DC in a 4:1 ratio (T cell:DC) for four days without additional stimuli. After four days of culture, cells were harvested and stained as described in a later section.

### Staining and flow cytometry analysis of T cells

Following co‐culture with the respective DC, T cells were stained for surface and intracellular markers using CD3 Pecy7‐A (clone SK7), CD25 BV421‐A (clone BC96), and IFNγ PerCP‐Cy5.5 (clone B27) and IL10 APC‐A (clone JES3‐pD7) (all from BioLegend), CD4 FITC‐A (clone RPA‐T4), CD8 APC‐H7‐A (clone SK1) (all from BD Biosciences). For the T cells used in proliferation assay, the cells were stained for surface markers: CD3 Pecy7‐A (clone SK7), CD4 FITC‐A (clone RPA‐T4), and CD8 PE‐A (clone SK1). Live/dead fixable dead cell stain kit Aqua‐A (Life Technologies) was included in all panels. For the intracellular staining of IFNϒ and IL10, monensin (BD Biosciences) was added to the co‐culture 5 h before analysis and then cells were fixed and permeabilized prior to intracellular staining. Corresponding isotype‐matched antibodies were used as negative controls. Detailed gating strategies can be found in Supplementary Figure S1B. The flow cytometry analysis was performed as mentioned above.

### Cytokine analysis (ELISA)

Levels of IL6, IL10, IL12, IL23, and TGFβ1, in the DC culture medium and levels of IL2, IFNγ, IL4, IL17, IL22, and IL10 in the DC:T cell culture media were measured with enzyme‐linked immunosorbent assays (ELISA) kits (MabTech, Nacka, Sweden). In short, plates were coated for 24 h, washed and blocked with BSA 0.1% v/v in PBS with 0.05% Tween. The DC supernatant was added and incubated overnight. Biotin labeled secondary antibodies and streptavidin‐ALP were then added prior to the phosphate substrate (Sigma–Aldrich). The optical density was determined using a micro‐plate reader (Molecular Devices Corp) set at 405 nm. Results were analyzed using SoftMax Pro 5.2 rev C (Molecular Devices Corp).

### Statistics

To assess differences between different bacteria‐conditioned DC in both pre‐ and post‐stimulation with LPS, the obtained raw data was log‐transformed. The normal distribution of the log transformed was tested using D'Agostino–Pearson Omnibus or Shapiro–Wilk normality test. If the data pass the normality test, the log‐transformed data were analyzed with one‐way ANOVA. If differences among groups were significant, Bonferroni multiple comparisons‐test was performed to control for multiple comparisons and confirm the *p*‐values between paired groups. If the data were not normally distributed, Kruskal–Wallis ANOVA was used to investigate differences among the groups. When significant, the Mann–Whitney U‐test was further used to investigate differences between groups. All statistical computations were performed using GraphPad Prism software V6 (La Jolla, CA).

## Results

### *Lactobacillus reuteri*‐CFS, but not S. aureus‐CFS, has a significant impact on DC differentiation

**Figure 1:**
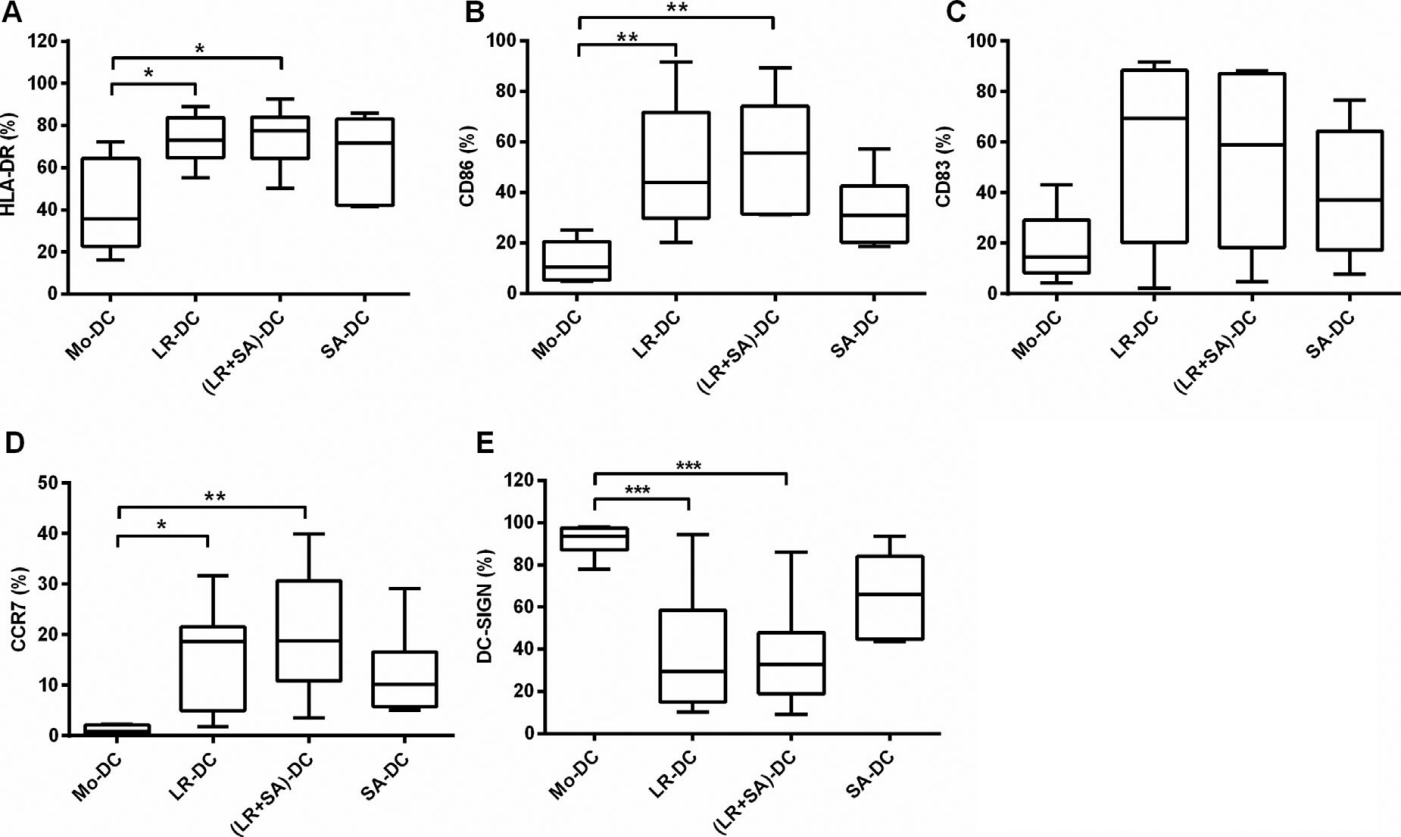
The presence of *Lactobacillus reuteri‐CFS* during the differentiation of DC influences the surface expression of DC markers associated with differentiation, antigen‐presentation, co‐stimulation, and T‐cell interaction. Box plot graphs (A–E) show the percentage of CD11c^+^ CD14^−^ cells expressing HLA‐DR (A) CD86 (B), CD83 (C), CCR7 (D), and DC‐SIGN (E) within the Mo‐DC, LR‐DC, (LR + SA)‐DC, and SA‐DC populations at day 6. Boxes extend data values from the 25th to 75th percentiles, with the central line are plotted at the median and extend from min to max. (*N* ≥ 6, ***p < 0.001, **p < 0.01, and *p < 0.05.

We investigated the expression of molecules associated with DC differentiation, antigen‐presentation, co‐stimulation, and T‐cell interactions in the generated DC after six days in culture with or without the presence of bacteria‐CFS. In general, all DC cultures (CD14^−^CD11c^+^ cells) showed a high degree of purity (Supplementary Fig. S1A). In DC generated in the presence of *L. reuteri*‐CFS, there was a significant increase in the percentages of cells expressing HLA‐DR and CD86 as well as in the relative expression of these markers, together with an increase in relative expression of CD83 (Fig. 1A–C, Supplementary Fig. S2 and Table S1). *Staphylococcus aureus*‐CFS‐generated DC also up‐regulated these markers, although not to a significant level. Further, the expression of CCR7, a lymphoid migration chemokine receptor, was also highly up‐regulated in DC generated in the presence of *L. reuteri*‐CFS (Fig. 1D, Supplementary Fig. S2 and Table S1). In contrast, DC‐SIGN, a molecule involved in pathogen recognition as well as DC–T cell interactions, was down‐regulated by *L. reuteri*‐CFS during DC differentiation (Fig. 1E, Supplementary Fig. S2 and Table S1). Although it affect the percentage of positive cells, *S. aureus*‐CFS tended to influence CCR7 and DC‐SIGN expression level in a similar fashion as *L. reuteri*‐CFS (Fig. 1D, E and Supplementary Table S1). The simultaneous presence of *L. reuteri*‐CFS and *S. aureus*‐CFS during DC differentiation did not result in any synergy, but were very similar to the *L. reuteri*‐CFS‐induced response throughout.

**Figure 2:**
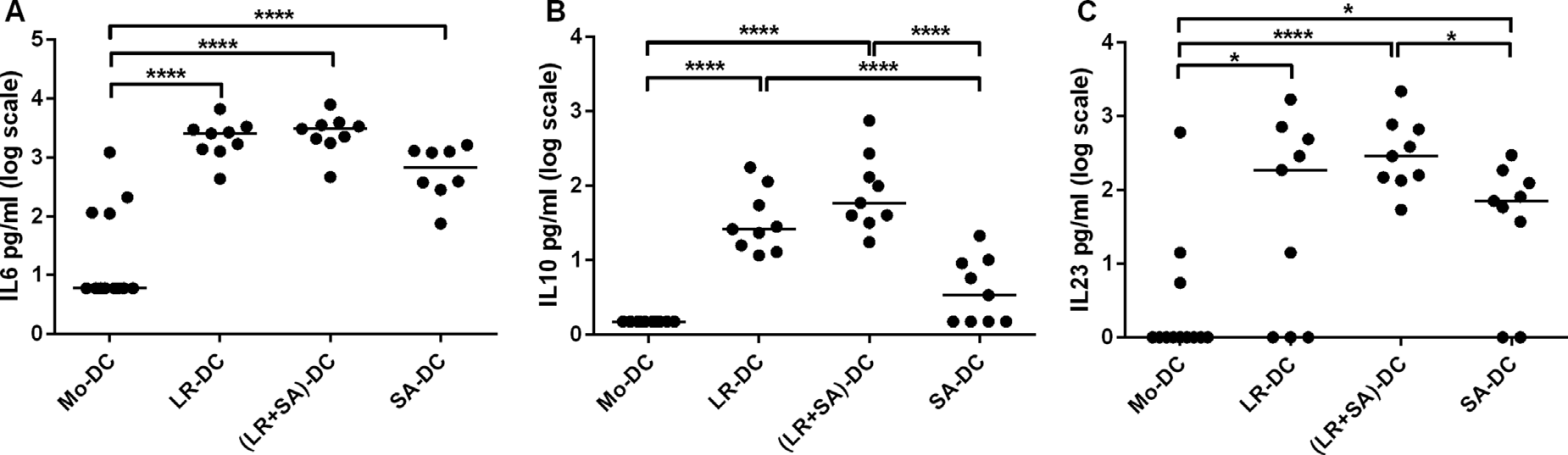
*Lactobacillus reuteri*‐CFS exposure during DC differentiation increases IL‐6, IL‐10, and IL‐23 level in the culture supernatant. Dot plot graph showing cytokines (IL6 (A), IL23 (B), and IL10 (C)) levels measured in the cell free supernatant of DC generated in the presence of bacteria‐CFS. Supernatant from conventional generated Mo‐DC was used as a control. *N* > 8, ***p < 0.001, **p < 0.01, and *p < 0.05.

Next, we measured the cytokine levels in the DC‐supernatants on day 6. DC exposed to *L. reuteri*‐CFS during their differentiation showed a prominent production of IL6, IL10, and IL23 (Fig. 2A–C). For *S. aureus*‐CFS exposure, the effect was less pronounced, but the levels of IL6 and IL23 differed significantly from that of untreated Mo‐DC (Fig. 2A–C). The levels of TGFβ1 were high in all cultures, and not affected by bacterial exposure during differentiation (data not shown). When both bacteria‐CFS were used simultaneously during maturation, the pattern was identical to that of *L. reuteri*‐CFS alone (Fig. 2A–C).

### DC generated in the presence of *L. reuteri*‐CFS exhibit decreased phagocytic ability

**Figure 3:**
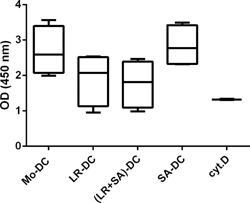
DC generated in the presence of *Lactobacillus reuteri*‐CFS exhibit decreased phagocytic ability. Box plot depicting the OD values, which correlates with the level of enzyme labeled *Escherichia coli* particle taken up by DC generated in the presence of bacteria‐CFS. MoDC treated with cytochalasin D, a phagocytosis inhibitor, were included as a negative control. Boxes extend data values from the 25th to 75th percentiles, with the central line are plotted at the median and extend from min to max. *N* = 4.

To assess the phagocytic ability of these DC populations, we co‐incubated the DC with enzyme‐labeled *E. coli* particles and investigated their uptake potential. *Lactobacillus reuteri*‐CFS‐generated DC were less efficient at internalizing *E. coli* particles than Mo‐DC, although not statistically significant, while *S. aureus*‐CFS*‐*generated DC were as efficient as regular Mo‐DC (Fig. 3). The effect of *L. reuteri* seemed to be dominant as co‐treatment with *L. reuteri*‐CFS and *S. aureus*‐CFS showed the same tendency of reduced antigen uptake, as did *L. reuteri*‐CFS alone (Fig. 3).

### The DC secretory profile following LPS stimulation is highly influenced by *L. reuteri*‐CFS

**Figure 4:**
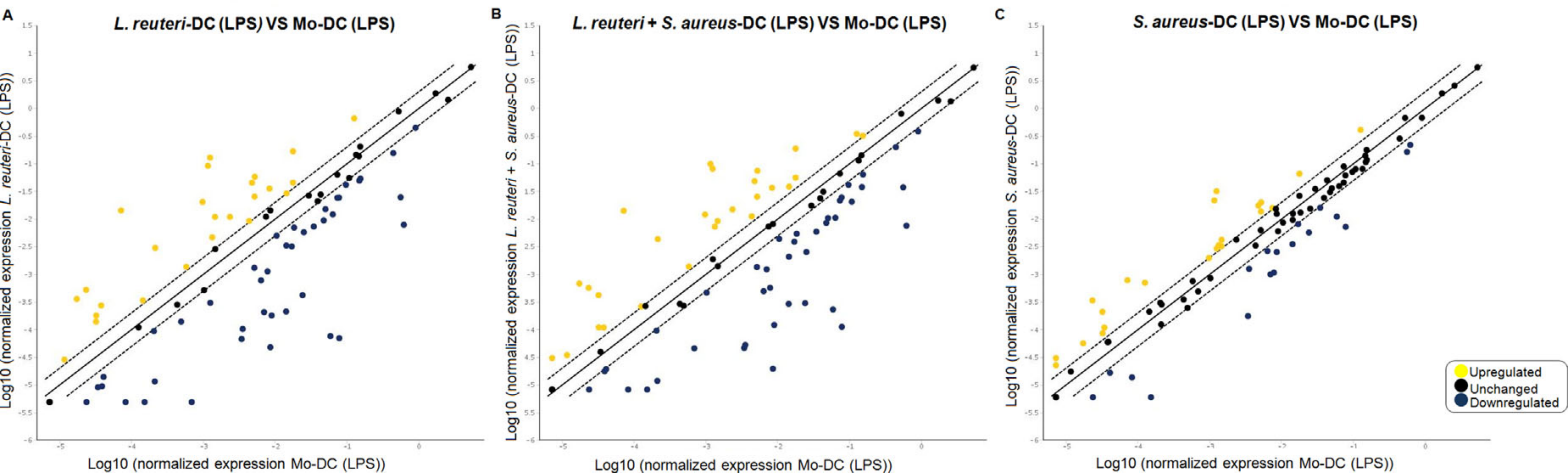
*Lactobacillus reuteri*‐CFS has a major impact on DC gene expression following LPS‐induced maturation. RT^2^ PCR arrays were performed on the RNA extracted from the LR‐DC, (LR + SA)‐DC, SA‐DC, and Mo‐DC stimulated with LPS for 24 h. Scatter plots shows the fold change of the gene expression of the LR‐DC (A), (LR + SA)‐DC (B), SA‐DC (C) in comparison to that of the Mo‐DC. Up‐regulated genes are shown in yellow, down‐regulated genes are shown in blue and unchanged (<2 fold change) genes in black.

In order to investigate whether the presence of bacterial factors during DC differentiation also had an impact on DC responses, the gene expression profiles of the generated DC post‐stimulation with LPS were assessed using RT^2^ PCR array. DC that had differentiated in the presence of *L. reuteri*‐CFS differed more in comparison to control Mo‐DC upon stimulation with LPS, than DC that were differentiated in the presence of *S. aureus*‐CFS (Fig. 4A vs. C). The pattern of the gene expression of the LPS matured DC generated in the presence of both *L. reuteri*‐CFS and *S. aureus*‐CFS resembled that of the *L. reuteri*‐CFS‐generated‐DC (Fig. 4B), again showing the dominant effect of *L. reuteri* over *S. aureus* in influencing the DC response.

In general, the presence of *L. reuteri*‐CFS during DC differentiation resulted in a profound reduction in mRNA expression following LPS‐maturation for cytokines and chemokines. This was evident also in the cultures where both *L. reuteri*‐ and *S. aureus*‐CFS were added, but it was much less pronounced in DC differentiated with *S. aureus*‐CFS alone (Supplementary Table S2). Of note, LPS‐stimulation resulted in a very strong reduction of mRNA expression in *L. reuteri*‐CFS‐generated DC for CCL19, CCL5, CCL7, CSF2, CXCL1, CXCL10, IFNG, IL10, IL12A, IL12B, IL6, and CXCL8, not seen in *S. aureus*‐CFS‐generated DC. In contrast, gene expression for CCL13, IL‐16, and TGFβ was enhanced by bacteria‐CFS, in particular for *L. reuteri*‐CFS‐generated DC.

The mRNA expression of several chemokine receptors and molecules related to antigen presentation were up‐regulated in LPS‐stimulated DC exposed to *L. reuteri* during differentiation (Supplementary Table S2).

**Figure 5:**
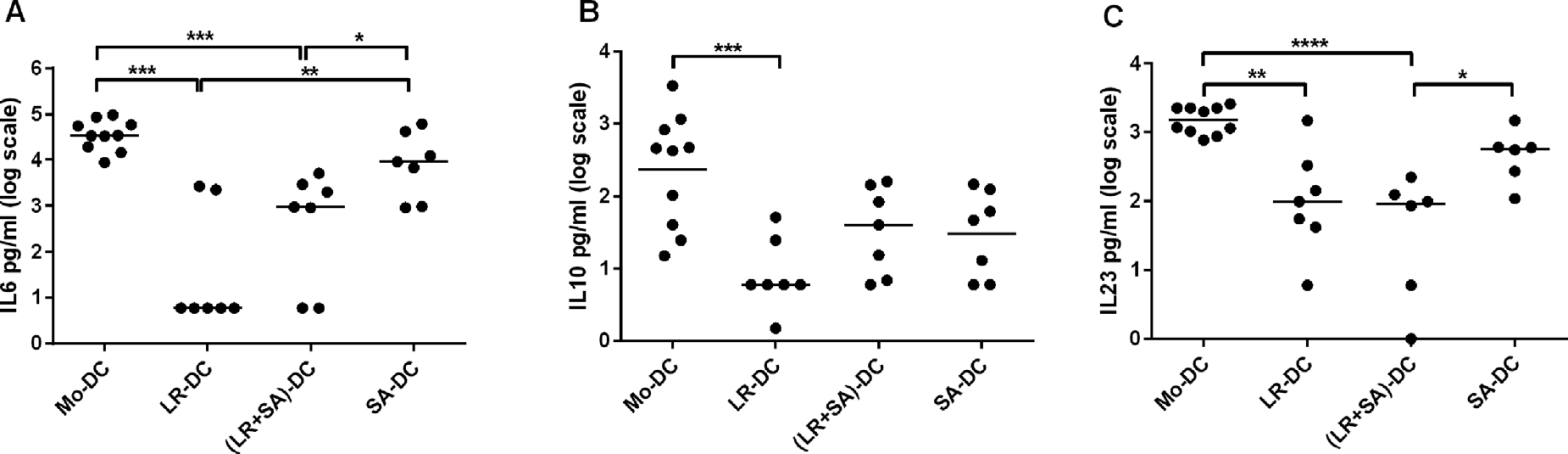
*Lactobacillus reuteri*‐CFS‐generated DC have a diminished cytokine production upon LPS stimulation. DC generated with bacteria‐CFS were further stimulated with LPS for 24 h. Dot plot showing the level of IL6 (A), IL23 (B), and IL10 (C) in culture supernatant from LR‐DC, (LR + SA)‐DC, SA‐DC, and Mo‐DC post‐stimulation with LPS was measured by ELISA. *N* > 6, ***p < 0.001, **p < 0.01, and *p < 0.05.

In line with the gene expression data, IL6, IL10, and IL23 were profoundly less secreted in the culture supernatants of the LPS‐treated *L. reuteri*‐CFS‐generated DC (Fig. 5A–C); however, we could not detect any differences in TGFβ production between the different DC groups (data not shown). LPS‐stimulated *S. aureus*‐CFS‐generated DC also had a tendency toward a lower IL6, IL10, and IL23 production compared with Mo‐DC, but it was not significant (Fig. 5A–C).

The reduced cytokine responses for *L. reuteri‐*CFS‐generated DC were not due to a lack of maturation following LPS exposure, as the expression of HLA‐DR, CD86, and CCR7 expression was similar in all DC groups, regardless of bacterial exposure during differentiation (Supplementary Fig. S3, S4, and Table S3).

### *Lactobacillus reuteri* exposure during DC differentiation has limited effects on T cell cytokine production

**Figure 6:**
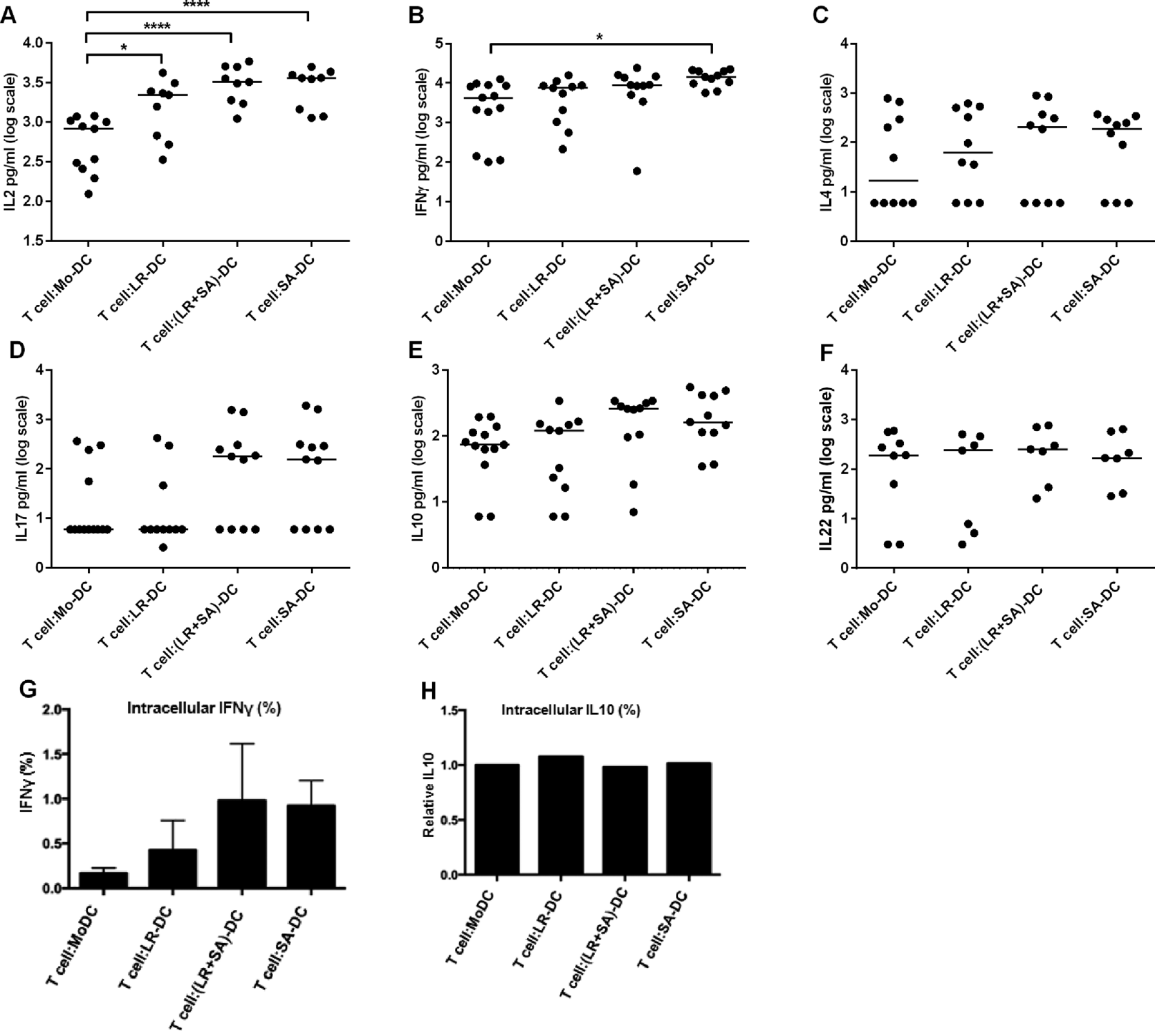
DC generated in the presence of *Staphylococcus aureus*‐CFS are efficient T cell activators in the absence of additional T cell stimulation. DC generated in the presence of bacteria‐CFS were stimulated with LPS and subsequently co‐cultured with T cells for 24 h. Dot plot showing the level of IL2 (A), IFNγ (B), IL4 (C), IL17 (D), IL10 (E), and IL22 (F) in culture supernatant from T cells co‐cultured with LPS matured Mo‐DC, LR‐DC, (LR + SA)‐DC, and SA‐DC was measured by ELISA. Bar graph showing %intracellular IFN‐γ (G) and IL10 (H) of CD4+ T cells. *N* > 7, ***p < 0.001, **p < 0.01, and *p < 0.05.

To evaluate the functional potential of the bacteria‐CFS‐generated DC, the different DC were stimulated with LPS and then co‐cultured with autologous T cells, without adding any T cell‐specific stimulation. The DC‐mediated T cell activation was evaluated by cytokine production in the supernatants of the co‐cultures using ELISA. All bacteria‐CFS‐generated DC were better at inducing IL2 production in T cells than were control Mo‐DC (Fig. 6A), but only *S. aureus*‐CFS‐generated DC could induce an IFNγ response in T cells that differed from that induced by Mo‐DC (Fig. 6B). This was confirmed by intracellular staining of IFNγ in T cells (Fig. 6G and Supplementary Fig. S5A). Although the results did not reach significance, there was also a tendency of increased levels of IL4 and IL17 when the T cells were co‐cultured with LPS‐matured *S. aureus*‐CFS‐generated DC (Fig. 6C, D). There were no obvious differences in the levels of IL10 and IL22 (Fig. 6E, F), confirmed for IL10 by intracellular staining (Fig. 6H and Supplementary Fig. S5B). There were no major differences in the viability of the T cells among the co‐cultures (data not shown).

To assess whether there was a need for *S. aureus*‐exposure already at DC generation to induce T cell cytokine production, the Mo‐DC were conditioned with bacteria‐CFS and co‐cultured with T cells after extensive washing of the DC. *Staphylococcus aureus*‐CFS‐pre‐treated Mo‐DC efficiently induced T cell proliferation and IL2, IFNγ, and IL10 production in the co‐cultures (Supplementary Fig. S6A–H). As expected, *L. reuteri*‐CFS‐conditioning of Mo‐DC had no effects on the T cell cytokine production.

## Discussion

In both humans and rodents, monocytes migrate into the gut driven by different signals 24, 25. What drives monocyte differentiation into mononuclear phagocytes (DC and macrophage) in the intestinal mucosa remains to be further verified. Most previous studies describing bacteria‐mediated effects on DC functions have used murine models and explored the microbe‐mediated effects on already generated DC 26, 27, 28, 29. Here, we designed an in vitro experimental system to study the effects of two gut microbes that are common in the neonatal gut, *L. reuteri* and *S. aureus*, on the differentiation of monocytes to DC. Further we measured cytokine responses following co‐cultures of these DC and unstimulated autologous T cells in vitro.

We clearly show that lactobacilli are a primary source of the signal in driving a gut DC phenotype. with a tolerogenic potential. The DC generated in the presence of *L. reuteri*‐CFS, up‐regulated maturation markers (HLA‐DR, CD86, CD83, and CCR7); characteristics similar to those of intestinal DC 14, 30. DC without this classical gut‐DC phenotype has been shown to exacerbate disease in a colitis model 30, a scenario that can be reversed by lactobacilli 31, 32. Also in humans, an inflammatory setting in the gut alters the DC phenotype. DC isolated from ulcerative colitis patients have increased expression of DC‐SIGN—a C‐type‐lectin receptor—and decreased expression of CD83 and CD86, a phenotype that could partially be restored by conditioning with serine–threonine peptide secreted by *L*. *plantarum* 33. In addition, Ara h1, a major peanut allergen, can bind to DC‐SIGN and drive allergy‐associated Th2 responses 34. Our results indicate the potential of *L. reuteri* to modulate the DC‐SIGN expression on DC and thereby possibly avert pathological conditions. Indeed, we and others have shown that colonization with lactobacilli early in life seems to decrease the risk for future allergy development 18, 20, 35.

The *L. reuteri*‐CFS‐generated DC were also less phagocytic, which might be due to the decrease in DC‐SIGN expression that is known to have a role in antigen capture 36, 37. This might be important to avoid the induction of harmful immune responses in the absence of infection, a situation similar to our experimental model. Indeed, gut‐DC have a decreased phagocytic ability 12.

Overall, the presence of bacteria‐CFS during DC differentiation induced IL6 and IL23, but *L. reuteri*‐CFS was more efficient in inducing IL10 than *S. aureus*‐CFS. Following LPS stimulation of the generated DC, we observed that *L. reuteri*‐CFS‐generated DC had a markedly altered secretory profile compared with LPS‐stimulated control Mo‐DC, but also in relation to *S. aureus*‐CFS‐generated DC. We noted an abrogated IL6, IL10, and IL23 production, confirmed both by gene expression and protein production. This could possibly be attributed to the observed down‐regulation of NFκB expression by *L. reuteri*‐CFS‐generated DC, as NFκB activation is important for the expression of these cytokines 38, 39, 40. Although we did not observe a difference at the protein level, TGFβ gene expression was higher for *L. reuteri*‐CFS‐generated DC than for *S. aureus*‐CFS‐generated DC upon stimulation with LPS. This further indicates the role of *L. reuteri*‐CFS in promoting the generation of regulatory DC. In the intestine, with its high load of microbial challenge, development of tolerogenic DC is important.

As *L. reuteri*‐CFS‐generated DC were significantly less responsive to LPS stimulation than control Mo‐DC and *S. aureus*‐CFS‐generated DC, it could be argued that *L. reuteri*‐CFS‐generated DC are exhausted and then become refractory to the LPS stimulation. However, the *L. reuteri*‐CFS‐generated DC did not display an altered surface phenotype following LPS stimulation. Moreover, the *L. reuteri*‐CFS‐generated DC‐induced IL2 production by the T cells. Further, when DC are exhausted, they preferentially drive Th2 responses 41, a characteristic that we did not observe in our T cell:DC co‐cultures. It has also been demonstrated that DC‐derived thrombospondin 1 acts as a negative regulator of DC cytokine production in situations where DC are exhausted 42. Here, we see a strong down‐regulation of thrombospondin 1 gene expression in *L. reuteri*‐CFS‐generated DC, arguing against an exhausted phenotype (see Supplementary Table S2, THBS1).

Although *S. aureus*‐CFS exposure had less pronounced effects on the differentiating DC than had *L. reuteri*‐CFS, *S. aureus*‐CFS‐generated DC were more efficient in activating T cells in a non‐inflammatory setting. *Staphylococcus aureus*‐CFS‐generated DC were able to induce cytokine production in autologous T cells without any additional T cell activation, significant for IL2 and IFNγ and a tendency also for IL17. *Staphylococcus aureus* produces different staphylococcal enterotoxins (SE) and toxic shock syndrome toxin‐1, which all have superantigenic properties. Superantigens drive uncontrolled T cell activation by non‐specifically linking MHC class II on APC and TCR on T cells, which might in certain cases lead to septic shock. We have recently demonstrated that the *S. aureus*‐CFS used in our study, potently induces FOXP3^+^ cells and promotes a diverse phenotype of these cells with production of both regulatory (IL10) and pro‐inflammatory (IFNγ and IL17) cytokines in a partly monocyte‐dependent manner 43. In a mouse model, DC isolated from SE treated mice tend to be more efficient in priming FOXP3 expression and gut homing phenotype (α4β7) on T cells compared to DC from sham‐fed mice 44. In our model, it is likely that SE have bound MHC on the DC that are capable of activating T cells, even though the CFS was added just once at day 3 of DC generation and that substantial washing of the DC was performed before co‐culture. However, we have previously shown that this *S. aureus*‐CFS induces inflammatory responses by intestinal epithelial cells, which might indicate its potential to use other mechanisms to activate cells than via the obvious MHC–TCR interaction 22.

*Staphylococcus aureus*‐CFS also had an impact on the T cell stimulatory capacity of already generated Mo‐DC, as *S. aureus*‐CFS‐treated Mo‐DC potently induced T cell proliferation and cytokine production. This is interesting in the context of the gut, where the DC are able to sample luminal antigen. Regardless of the micro‐environment during their differentiation and maturation, the DC then can capture and present *S. aureus*‐produced factors that can shape the response of the intestinal T cells.

Recently, it has been shown that breast‐feeding plays a vital role in the early colonization with *S. aureus* and different lactobacilli, and that the nutrients in breast milk helps in maintaining these microbes in the infant gut 45. We have previously shown that lactobacilli dampen *S. aureus*‐CFS‐induced PBMC responses 22. Further, we have recently observed that the dampening effect might be mediated in different ways depending on the responding immune cell (*Johansson, Björkander et al. unpublished observation*). Similarly, in the differentiation of monocytes to DC, the presence of both *S. aureus*‐CFS and *L. reuteri*‐CFS resulted in a DC population that has a distinct phenotype. These DC shared certain features of the *L. reuteri*‐CFS‐generated DC (e.g., tolerogenic phenotype, less responsive to LPS stimulation, and less phagocytic ability) and certain features of *S. aureus*‐CFS‐generated‐DC (e.g., efficient in inducing T cell response). This indicates that in our setting, *S. aureus* was not able to interfere with the *L. reuteri* imprinted phenotype of DC, but also that exposure to lactobacilli during differentiation is not enough to abolish *S. aureus*‐mediated DC activation of T cells. This is in contrast to another study where co‐stimulation with *S. aureus* and *L. reuteri* inhibits DC response to *S. aureus* 46, possibly explained by the use of different strains and whole bacteria instead of CFS in this other study.

Due to their accessibility and the possibility to generate a high number of cells in vitro, manipulation of Mo‐DC in vitro and retransferring them into humans has been recommended for individualized immunotherapy in several diseases 47. However, DC have tissue‐specific properties. Mimicking the gut micro‐environment during the generation of Mo‐DC by including microbial signals might serve as a future approach to be used to generate gut‐specific DC when treating diseases in the gastro‐intestinal tract. The presence of *L. reuteri‐*CFS triggered IL6 and IL23 production from the DC. In the context of the gut, these cytokines have a role in bolstering the host immunity 48, 49, 50. This can indicate that beneficial effects of probiotics are not limited to dampening immune response, but also to support the host immune system in the defense against pathogens. In fact, some strains activate the immune system and contribute by initiating defense mechanisms 51.

In conclusion, our data underline that signals from different microbes are of importance for shaping of the DC population, in line with the concept that the gut DC have to develop an ability a tolerogenic phenotype, but yet be responsive to challenges in order to keep the homeostasis of the gut.

## Supporting information

Additional supporting information may be found in the online version of this article at the publisher's web‐site.

**Figure S1**. The gating strategy for DC (A) and T cells (B).
**Figure S2**. The presence of L. reuteri‐CFS during the differentiation of DC increased the surface expression of HLA‐DR, CD86, CD83 and CCR7.
**Figure S3**. LPS stimulation did not hamper expression of maturation markers on L. reuteri‐CFS‐generated DC.
**Figure S4**. The generated DC did not show differences on the surface expression of HLA‐DR, CD86, and CCR7, upon stimulation with LPS.
**Figure S5**. DC generated in the presence of *S. aureus*‐CFS induce IFNγ production by T cell.
**Figure S6**. *S. aureus*‐CFS conditioned Mo‐DC induce T cell proliferation and cytokine production.
**Table S1**. The fold change in the MFI of the surface markers of bacteria‐CFS‐generated‐DC compared to that of the Mo‐DC in culture medium.
**Table S2**. mRNA expression of the 84 genes investigated using RT2 PCR array. Results show fold regulation of bacteria‐CFS‐generated‐DC compared to that of Mo‐DC, following LPS stimulation.
**Table S3**. The fold change in the MFI of the surface markers of LPS‐stimulated bacteria‐CFS‐generated‐DC compared to that of the LPS‐stimulated Mo‐DC.
