# Supplementary material for: Lactobacillus reuteri and Staphylococcus aureus differentially influence the generation of monocyte‐derived dendritic cells and subsequent autologous T cell responses
Source: Immun Inflamm Dis. 2016 Jul 29;4(3):315–26. doi: 10.1002/iid3.115 (PMC5004286; doi:10.1002/iid3.115)
Supplement: Supplementary file 1 — Figure S1. The gating strategy for DC (A) and T cells (B). Figure S2. The presence of L. reuteri‐CFS during the differentiation of DC increased the surface expression of HLA‐DR, CD86, CD83 and CCR7. Figure S3. LPS stimulation did not hamper expression of maturation markers on L. reuteri‐CFS‐generated DC. Figure S4. The generated DC did not show differences on the surface expression of HLA‐DR, CD86, and CCR7, upon stimulation with LPS. Figure S5. DC generated in the presence of S. aureus‐CFS induce IFNγ production by T cell. Figure S6. S. aureus‐CFS conditioned Mo‐DC induce T cell proliferation and cytokine production. Table S1. The fold change in the MFI of the surface markers of bacteria‐CFS‐generated‐DC compared to that of the Mo‐DC in culture medium. Table S2. mRNA expression of the 84 genes investigated using RT2 PCR array. Results show fold regulation of bacteria‐CFS‐generated‐DC compared to that of Mo‐DC, following LPS stimulation. Table S3. The fold change in the MFI of the surface markers of LPS‐stimulated bacteria‐CFS‐generated‐DC compared to that of the LPS‐stimulated Mo‐DC. [file IID3-4-315-s001.docx]

Supplementary Figure 1


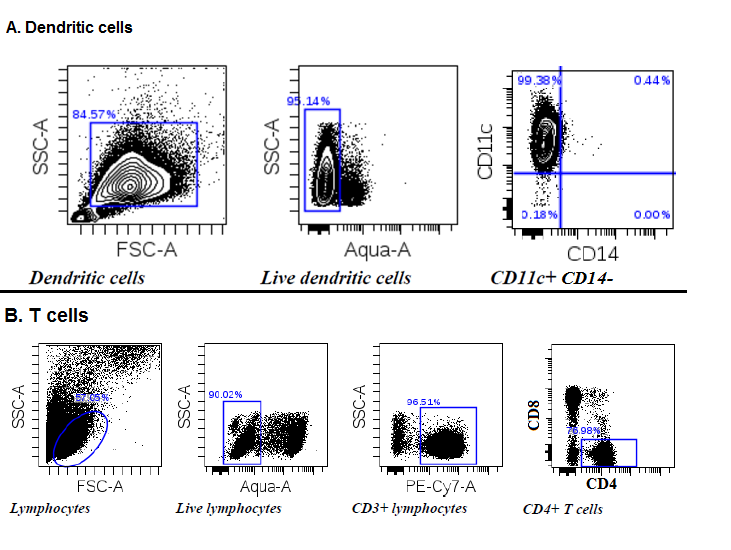


**Supplementary Figure 1. The gating strategy for DC (A) and T cells (B).** In supplementary Figure 1 A, from the forward scatter (FSC-A) and side scatter (SSC-A), dendritic cells were gated. Then to gate for the live DC, we gated Aqua (low) vs SSC-A. In the live DC population, we looked at CD11c vs CD14. In the gated CD11c+ CD14-, we looked the surface markers (HLA-DR, CD83, CD86, CCR7 and DC-SIGN) expression. In supplementary Figure 1 B, from the forward scatter (FSC) and side scatter (SSC-A), lymphocytes were gated. Live lymphocytes were then gated from aqua vs SSC-A. CD3+ lymphocytes were gated from CD3 vs SSC-A. CD4+ T cells were gated from CD4 vs CD8. In the CD4+ T cells, we looked at the intracellular IL10 and IFNγ.

Supplementary Figure 2


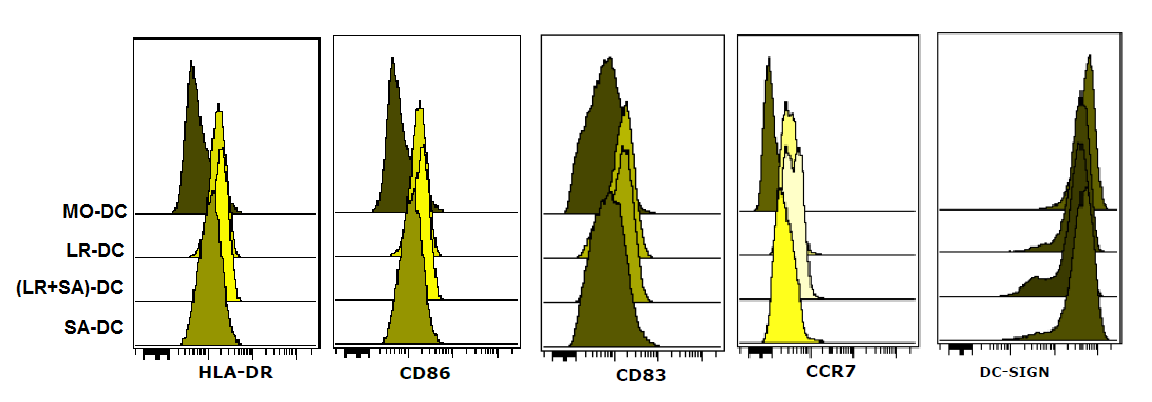


**Supplementary Figure 2. The presence of *L. reuteri-CFS* during the differentiation of DC increased the surface expression of HLA-DR, CD86, CD83 and CCR7.** Representative histogram showing the surface expression of HLA-DR, CD86, CD83, CCR7 and DC-SIGN of CD11c^+^ CD14^-^ Mo-DC, LR-DC, (LR+SA)-DC and SA-DC

Supplementary Figure 3


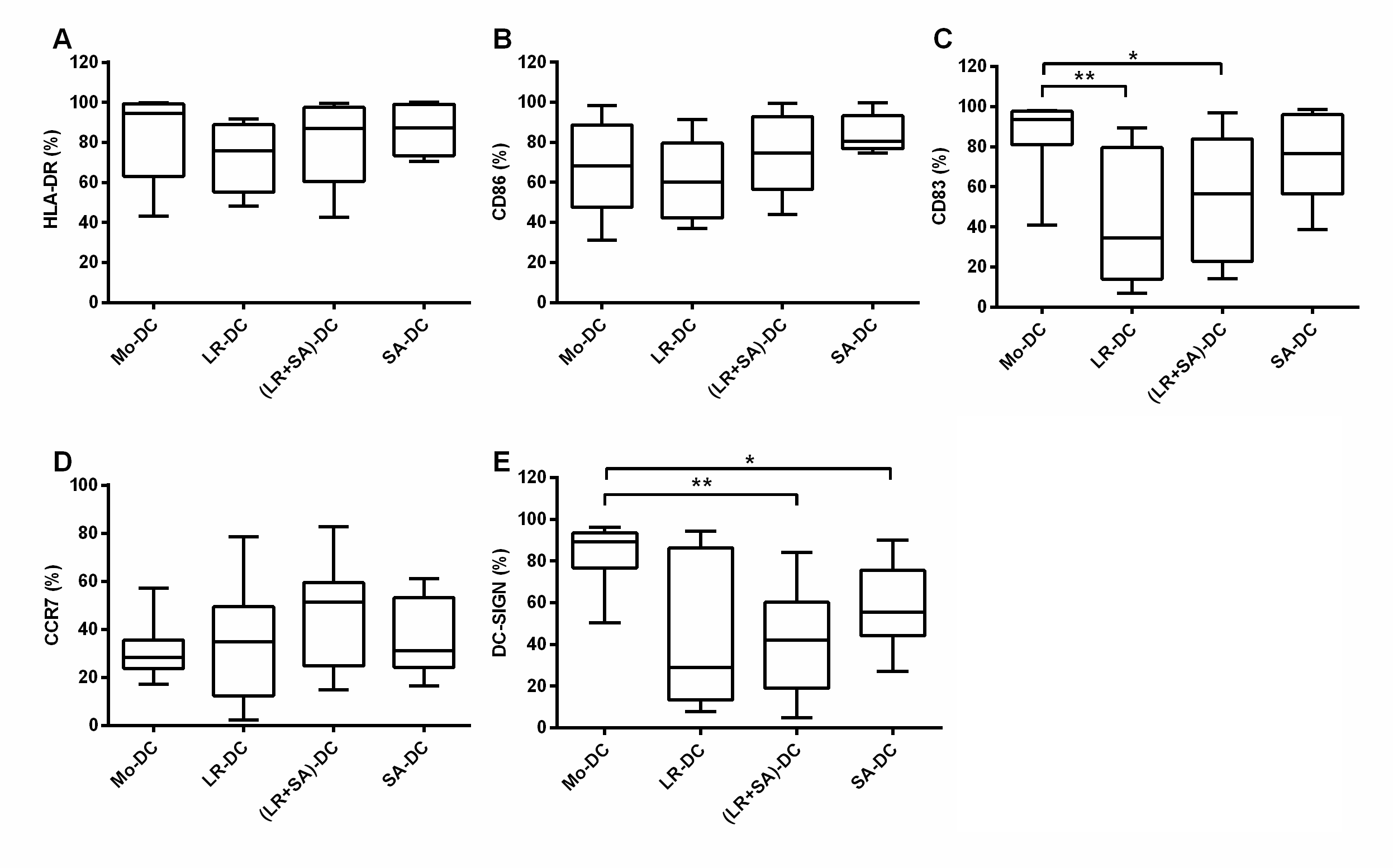


**Supplementary Figure 3. LPS stimulation did not hamper expression of maturation markers on *L. reuteri*-CFS-generated DC.** Mo-DC, LR-DC, (LR+SA)-DC and SA-DC were stimulated with LPS for 24hrs. Box plot graphs (A-E) show the percentage of CD11c^+^ CD14^-^ cells expressing HLA-DR (A), CD86 (B), CD83 (C), CCR7 (D) and DC-SIGN (E) in Mo-DC, LR-DC, (LR+SA)-DC and SA-DC post-stimulation with LPS. Representative heat map showing MFI fold changes of the surface markers HLA-DR, CD86, CD83, CCR7 and DC-SIGN of of CD11c^+^ CD14^-^LR-DC, (LR+SA)-DC and SA-DC post-treatment in comparison to culture medium is shown in (F). Boxes extend data values from the 25th to 75th percentiles, with the central line are plotted at the median and extend from min to max. N≥7, ***P < 0·001, **P < 0·01 and *P < 0·05.

Supplementary Figure 4


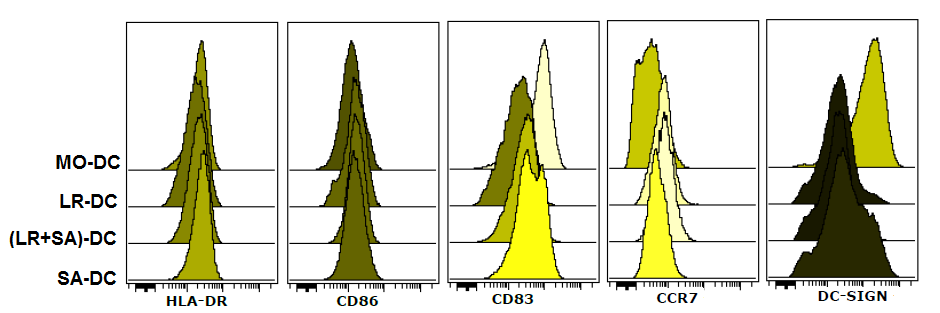


**Supplementary Figure 4. The generated DC did not show differences on the surface expression of HLA-DR, CD86 and CCR7, upon stimulation with LPS.** Representative histogram showing the surface expression of HLA-DR, CD86, CD83, CCR7 and DC-SIGN of CD11c^+^ CD14^-^ Mo-DC, LR-DC, (LR+SA)-DC and SA-DC stimulated with LPS

Supplementary Figure 5


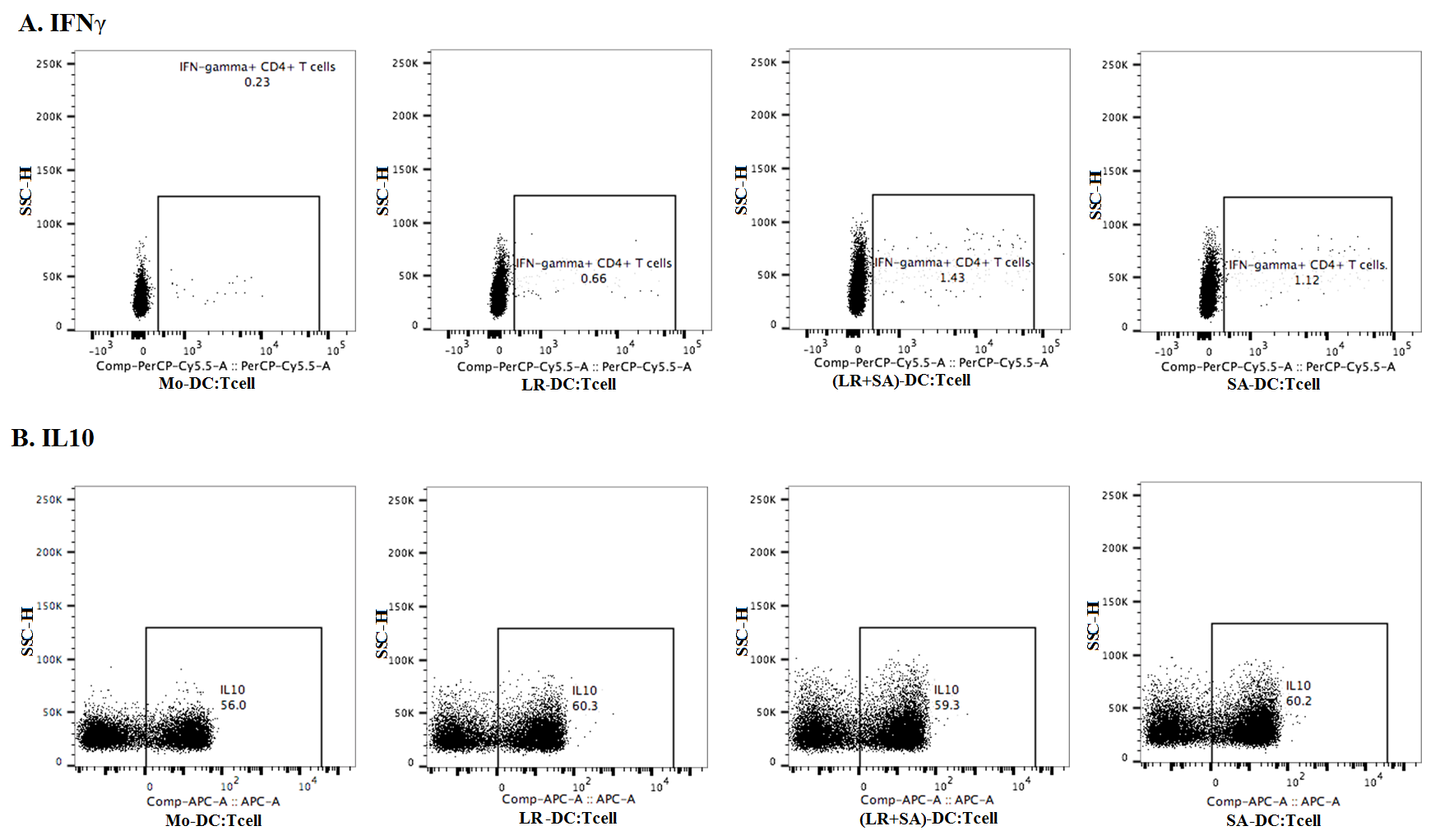


**Supplementary Figure 5. DC generated in the presence of *S. aureus*-CFS induce IFNγ production by T cell.** Flow cytometry analysis of %intracellular IFN-γ (A) and IL10 (B) of CD4+ T cells co-cultured with LPS activated Mo-DC, LR-DC, (LR+SA)-DC and SA-DC.





Supplementary Figure 6

**Supplementary Figure 6. *S. aureus*-CFS conditioned Mo-DC induce T cell proliferation and cytokine production.** For the proliferation assay, conventionally generated Mo-DC were pretreated with bacteria-CFS and then co-cultured with CellTrace^TM^ Violet stained T cells for 24hrs. (A) Representative plot showing a flow cytometry analysis of %proliferation of CD4+ T cells. (B) Bar graph showing %proliferation of CD4+ T cells. N=4. Conventional generated Mo-DC were pretreated with bacteria-CFS and then co-cultured with T cells for 24hrs. Dot plot showing the level of IL2 (C), IFNγ (D), IL4 (E), IL10 (F), IL17 (G) and IL22 (H) in culture supernatant from T cells co-cultured with Mo-DC, LR-DC, (LR+SA)-DC and SA-DC post-stimulation with LPS was measured by ELISA. N>7, ****P* < 0·001, ***P* < 0·01 and **P* < 0·05.

Supplementary Table 1: the fold change in the MFI of the surface markers of bacteria-CFS-generated-DC compared to that of the Mo-DC in culture medium

|  | **HLA-DR** | **CD86** | **CD83** | **CCR7** | **DC-SIGN** |
| --- | --- | --- | --- | --- | --- |
| **Mo-DC** | 1,00 | 1,00 | 1,00 | 1,00 | 1,00 |
| **LR-DC** | 2,88* (0,37-5,51) | 3,99** (0,95-8,86) | 3,52* (0,31-7,68) | 4,79** (1,94-9,12) | 0,29*** (0,14-0,34) |
| **(LR+SA)-DC** | 3,12* (1,12-5,44) | 3,95** (1,39-7,90) | 3,26 (0,90-7,44) | 5,73*** (2,67-12,18) | 0,24*** (0,10-0,22) |
| **SA-DC** | 2,51 (0,46-5,75) | 2,25 (1,16-3,73) | 1,93 (0,31-4,56) | 4,46* (2,17-6,42) | 0,55*** (0,21-0,87) |

Average fold increase (min-max) in MFI of 8 experiments. *=P<0,05, **=P<0,005, ***=P<0,0005

Supplementary Table 2: mRNA expression of the 84 genes investigated using RT2 PCR array. Results show fold regulation of bacteria-CFS-generated-DC compared to that of Mo-DC, following LPS stimulation.

|  | *Name* | *Fold regulation*  *LR-DC* | *Fold regulation*  *(L+S)-DC* | *Fold regulation*  *SA-DC* |  | *Name* | *Fold regulation*  *LR-DC* | *Fold regulation*  *(L+S)-DC* | *Fold regulation*  *SA-DC* |  | *Name* | *Fold regulation*  *LR-DC* | *Fold regulation*  *(L+S)-DC* | *Fold regulation*  *SA-DC* |
| --- | --- | --- | --- | --- | --- | --- | --- | --- | --- | --- | --- | --- | --- | --- |
| **Cytokines** | CCL11 | -1,46^B^ | 1,15^B^ | -1,19^B^ | **Cytokine receptors** | CCR1 | **4,68** | **6,39** | 1,81 | **Antigen presentation** | CD1A | **201,75^A^** | **198,97^A^** | **11,07^A^** |
|  | CCL13 | **5,26** | **2,76** | **3,25** |  | CCR2 | **5,64^A^** | **3,45** | **2,69** |  | CD1B | **79,70** | **86,01** | **18,88** |
|  | CCL16 | -1,46^B^ | 1,15^B^ | -1,19^B^ |  | CCR3 | **7,29^A^** | **2,92** | 1,59 |  | CD1C | **104,43** | **65,64** | **25,79** |
|  | CCL19 | **-1107,87^A^** | **-691,51^A^** | **-10,79** |  | CCR5 | **7,55** | **6,35** | **2,23** |  | CD1D | **4,40** | **13,24^A^** | **6,63^A^** |
|  | CCL2 | **-4,33** | **-9,87** | -1,63 |  | CSF1R | **2,14** | **2,58** | -1,30 |  | CD209 | **4,28** | **4,43** | 1,84 |
|  | CCL3 | **-4,70** | **-5,83** | **-2,15** |  | CXCR1 | **20,91^A^** | **39,85^A^** | **3,31** |  | CD28 | **22,73^A^** | **24,70^A^** | **14,61^A^** |
|  | CCL5 | **-22,53** | **-14,86** | **-3,41** |  | CXCR4 | -1,94 | **-5,22** | -1,34 |  | CD4 | 1,68 | -1,05 | 1,45 |
|  | CCL7 | **-49,30** | **-73,19^A^** | -1,48 |  | FLT3 | **-4,07** | 1,51 | **2,36** |  | CD40 | -1,55 | -1,38 | 1,17 |
|  | CCL8 | **-4,77** | **-2,84** | **-3,89** |  | ERBB2 | **2,49** | **3,00** | 1,51 |  | CD40LG | **-3,72** | 1,17 | **3,22** |
|  | CSF2 | **-18,05** | **-17,67** | -1,68 | **Signal transduction** | CDKN1A | **-2,84** | **-2,21** | -1,55 |  | CD74 | 1,09 | -1,23 | 1,09 |
|  | CXCL1 | **-177,73^A^** | **-434,62^A^** | **-3,37** |  | CEBPA | **11,21** | **14,38** | **3,86** |  | CD80 | **-3,33** | **-4,80** | -1,39 |
|  | CXCL10 | **-767,26^A^** | **-253,11^A^** | **-5,32** |  | CLEC4C | -1,46 | 1,15^B^ | -1,19^B^ |  | CD86 | **-2,86** | **-2,40** | -1,31 |
|  | CXCL12 | **-30,77** | **-18,30** | **-25,13** |  | FAS | **-5,29** | **-4,36** | **-2,10** |  | CD8A | **-4,07** | **-2,18** | 1,56 |
|  | CXCL2 | **-66,42** | **-48,29** | **-4,03** |  | IRF7 | **-8,13** | **-12,76** | **-2,40** |  | HLA-A | **-2,02** | **-2,34** | -1,33 |
|  | FLT3LG | -1,96 | **-2,18** | -1,19 |  | IRF8 | 1,48 | -1,02 | **2,13** |  | HLA-DMA | **2,58** | **3,17** | 1,48 |
|  | IFNG | **-139,45^A^** | **-14,86^A^** | -1,40 |  | ITGAM | **9,56** | **10,24** | **3,70** |  | HLA-DPA1 | 1,69 | 1,53 | 1,28 |
|  | IL10 | **-49,99^A^** | **-72,69^A^** | **-19,31^A^** |  | ITGB2 | **9,49** | **10,60** | **3,73** |  | TAPBP | -1,10 | -1,67 | 1,20 |
|  | IL12A | **-16,72** | **-9,94** | **-5,99** |  | LYN | **-3,02** | **-3,40** | -1,60 |  | THBS1 | **-33,67** | **-5,67** | **-6,97** |
|  | IL12B | **-34,14^A^** | **-66,42^A^** | **-2,79** |  | NFKB1 | **-5,22** | **-5,99** | -1,61 | **Other cell surface receptors** | CD2 | -1,13 | **2,11** | **5,65^A^** |
|  | IL16 | **14,19^A^** | **20,48^A^** | 1,35^A^ |  | PTPRC | **2,36^A^** | 1,87^A^ | 1,48^A^ |  | FCER1A | **21,06** | **12,44** | **2,04** |
|  | IL2 | -1,46^B^ | **4,28** | **4,28** |  | RELA | **-2,61** | **-3,37** | -1,40 |  | FCER2 | **2,04** | **2,69** | -1,14 |
|  | IL6 | **-57,03** | **-79,54** | **-4,20** |  | RELB | **-4,30** | **-6,84** | -1,48 |  | FCGR1A | **-3,49^A^** | -1,81 | -1,97 |
|  | CXCL8 | **-78,44** | **-81,78** | **-2,83** |  | STAT3 | **-3,17** | **-3,15** | -1,26 |  | LRP1 | **3,52** | **5,49** | **2,53** |
|  | MIF | -1,86 | -1,64 | -1,61 | **Antigen uptake** | CD44 | 1,08 | -1,18 | -1,66 |  | TLR1 | **2,37** | **2,41** | 1,31 |
|  | TGFB1 | **4,95** | **4,91** | **2,65** |  | CDC42 | -1,15 | -1,10 | 1,22 |  | TLR2 | **-6,89** | **-13,68** | **-7,27** |
|  | TNF | **-3,90** | **-3,77** | 1,23 |  | ICAM1 | **-3,00** | **-3,93** | -1,39 |  | TLR7 | **-2,16** | **-2,12** | 1,50^A^ |
|  | TNFSF11 | -1,46^B^ | 1,15^B^ | **3,18** |  | ICAM2 | -1,52 | -1,47 | -1,22 |  | TLR9 | **-2,90** | **-2,09** | **-2,43** |
|  |  |  |  |  |  | RAC1 | **-2,32** | **-2,32** | -1,36 |  | VCAM1 | 1,97 | -1,04 | **2,88** |
|  |  |  |  |  |  | TAP2 | **-5,01** | **-5,55** | -1,54 |  |  |  |  |  |

^A^ This gene’s average threshold cycle is relatively high (> 30) in either the control or the test sample, and is reasonably low in the other sample (< 30). These data mean that the gene’s expression is relatively low in one sample and reasonably detected in the other sample suggesting that the actual fold-change value is at least as large as the calculated and reported fold-change result.

^B^ This gene’s average threshold cycle is either not determined or greater than the defined cut-off (default 35), in both samples meaning that its expression was undetected, making this fold-change result erroneous and un-interpretable. Values in bold are considered biologically relevant difference (≥ 2 fold change).

Supplementary Table 3: the fold change in the MFI of the surface markers of LPS stimulated bacteria-CFS-generated-DC compared to that of the LPS stimulated Mo-DC

|  | HLA-DR | CD86 | CD83 | CCR7 | DC-SIGN |
| --- | --- | --- | --- | --- | --- |
| Mo-DC | 1,00 | 1,00 | 1,00 | 1,00 | 1,00 |
| LR-DC | 0,83 (0,44-1,19) | 1,04 (0,56-1,78) | 0,27** (0,08-1,05) | 1,23 (0,56-2,31) | 0,87 (0,11-4,59) |
| (LR+SA)-DC | 0,99 (0,62-1,52) | 1,27 (0,83-2,55) | 0,39** (0,12-1,67) | 1,66 (1,06-2,76) | 0,48* (0,08-2,10) |
| SA-DC | 1,21 (0,96-1,54) | 1,26 (0,91-1,69) | 0,56*** (0,29-0,93) | 1,30 (0,80-1,68) | 0,55 (0,20-0,91) |

Average fold increase (min-max) in MFI of 8 experiments. *=P<0,05, **=P<0,005, ***=P<0,0005
